# (3*S*,5*R*,6*S*)-Di­phenyl­methyl 1-oxo-6-bromo­penicillanate

**DOI:** 10.1107/S2414314620001431

**Published:** 2020-02-11

**Authors:** Krishnan Soundararajan, Velusamy Sethuraman, Kaliyaperumal Thanigaimani

**Affiliations:** a Department of Chemistry, Periyar Maniammai Institute of Science and Technology, Vallam-613 403, Thanjavur, Tamil Nadu, India; b Department of Chemistry, Government Arts College, Tiruchirappalli- 620 022, Tamil Nadu, India; Okayama University, Japan

**Keywords:** crystal structure, 6-bromo­penicillanate, β-lactamase inhibitor, hydrogen bond

## Abstract

In the title compound, C_21_H_20_BrNO_4_S, a key inter­mediate in the synthesis of the widely used β-lactamase inhibitor tazobactam, the five-membered thia­zolidine ring adopts an envelope conformation and the four-membered azetidine ring is in a distorted planar conformation. The crystal structure features C—H⋯O hydrogen bonds and a weak C—H⋯π inter­action.

## Abstract 2

In the title compound, C_21_H_20_BrNO_4_S, a key inter­mediate in the synthesis of the widely used β-lactamase inhibitor tazobactam, the five-membered thia­zolidine ring adopts an envelope conformation and the four-membered azetidine ring is in a distorted planar conformation.

## Graphical abstract

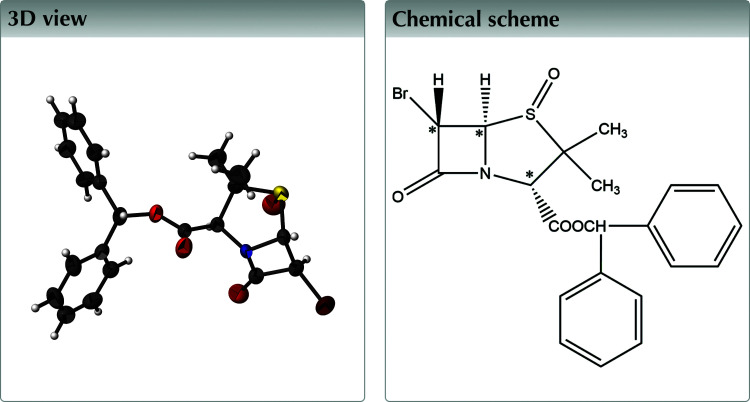

## Structure description

**Figure 1:**
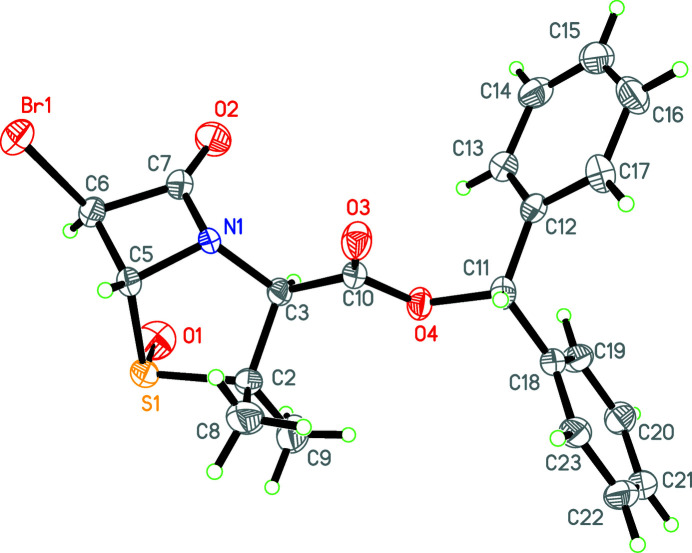
The mol­ecular structure of the title compound with atom labels and 50% probability displacement ellipsoids.

The title compound (Fig. 1 ▸) is a key inter­mediate for the synthesis of tazobactam, a widely used β-lactamase inhibitor (Bai *et al.*, 2001 ▸). The five-membered thia­zolidine ring (N1/C3/C2/S1/C5) adopts an envelope conformation, with an r.m.s deviation of 0.318 Å and a maximum deviation of 0.305 (1) Å for atom S1. The four-membered azetidine ring (N1/C5–C7) is in a distorted planar conformation, with an r.m.s deviation of 0.052 Å. The dihedral angle between the mean planes of these rings is 49.7 (2)°. The two phenyl rings of the di­phenyl­methyl group are inclined at an angle of 79.0 (2)°.

**Figure 2:**
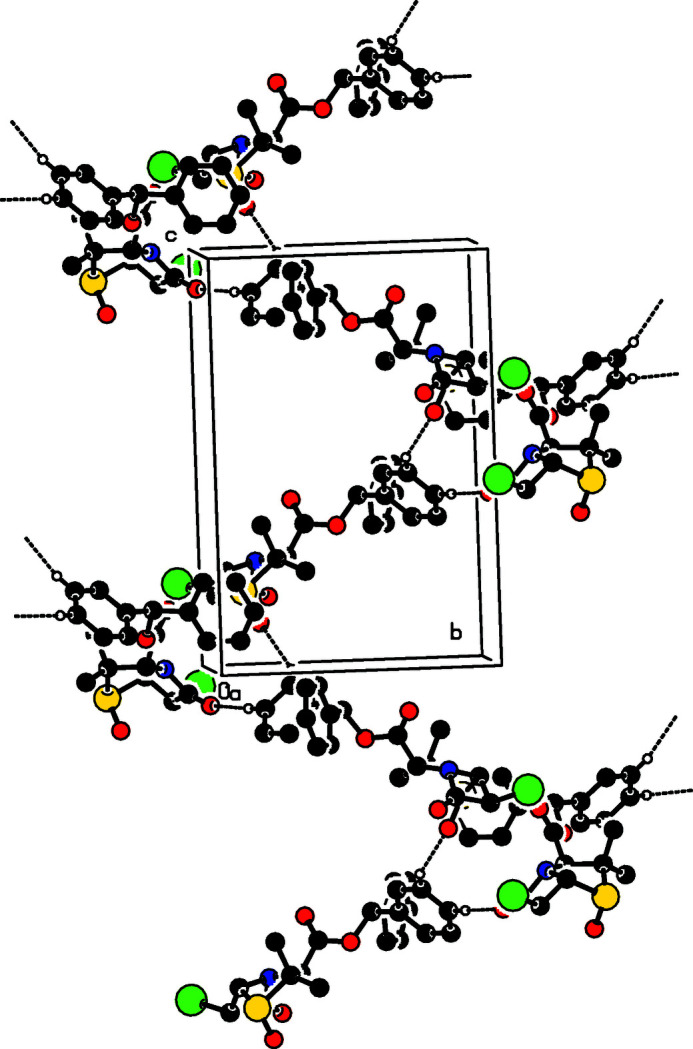
A partial packing diagram for the title compound. Dashed lines indicate the C—H⋯O hydrogen bonds.

In the crystal (Fig. 2 ▸), the mol­ecules self-assemble *via* C21—H21⋯O2 and C22—H22⋯O1 hydrogen bonds (Table 1 ▸), forming a three-dimensional network. Weak C—H⋯π inter­actions involving the C18–C23 ring also occur.

## Synthesis and crystallization

The title compound, which was a gift sample from Orchid Pharmaceutical Ltd, India, prepared according to the procedure of Xu *et al.* (2005 ▸), was dissolved in aceto­nitrile. It was heated over a water bath for few minutes and the resultant solution was allowed to cool. After a week, transparent yellow block-shaped crystals separated out.

## Refinement

Crystal data, data collection and structure refinement details are summarized in Table 2 ▸.

## Supplementary Material

Crystal structure: contains datablock(s) global, I. DOI: 10.1107/S2414314620001431/is4040sup1.cif

Structure factors: contains datablock(s) I. DOI: 10.1107/S2414314620001431/is4040Isup2.hkl

CCDC reference: 1981292

Additional supporting information:  crystallographic information; 3D view; checkCIF report

## Figures and Tables

**Table 1:** Hydrogen-bond geometry (Å, °) *Cg* is the centroid of the C18–C23 ring.

*D*—H⋯*A*	*D*—H	H⋯*A*	*D*⋯*A*	*D*—H⋯*A*
C21—H21⋯O2^i^	0.93	2.54	3.465 (5)	171
C22—H22⋯O1^ii^	0.93	2.55	3.375 (5)	148
C14—H14⋯*Cg* ^iii^	0.93	2.68	3.533 (5)	153

Symmetry codes: (i) 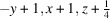 ; (ii) 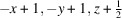 ; (iii) 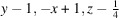 .

**Table 2:** Experimental details

Crystal data	
Chemical formula	C_21_H_20_BrNO_4_S
*M*_r_	462.35
Crystal system, space group	Tetragonal, *P*4_1_
Temperature (K)	293
*a*, *c* (Å)	11.076 (2), 16.478 (3)
*V* (Å^3^)	2021.4 (7)
*Z*	4
Radiation type	Mo *K*α
μ (mm^−1^)	2.16
Crystal size (mm)	0.30 × 0.25 × 0.20

Data collection	
Diffractometer	Bruker Kappa APEXII CCD
Absorption correction	Multi-scan (*SADABS*; Bruker, 2004 ▸)
*T*_min_, *T*_max_	0.563, 0.672
No. of measured, independent and observed [*I* > 2σ(*I*)] reflections	20917, 4005, 2553
*R*_int_	0.072
(sin θ/λ)_max_ (Å^−1^)	0.619

Refinement	
*R*[*F* ^2^ > 2σ(*F* ^2^)], *wR*(*F* ^2^), *S*	0.033, 0.083, 0.89
No. of reflections	4005
No. of parameters	255
No. of restraints	1
H-atom treatment	H-atom parameters constrained
Δρ_max_, Δρ_min_ (e Å^−3^)	0.20, −0.27
Absolute structure	Flack (1983 ▸)
Absolute structure parameter	0.016 (8)

Computer programs: *APEX2* and *SAINT* (Bruker, 2004 ▸), *SIR92* (Altomare *et al.*, 1993 ▸), *SHELXL2014* (Sheldrick, 2015 ▸) and *ORTEP-3 for Windows* (Farrugia, 2012 ▸).

## full crystallographic data

### Crystal data

**Table:**

C_21_H_20_BrNO_4_S	*D*_x_ = 1.519 Mg m^−^^3^
*M**_r_* = 462.35	Mo *K*α radiation, λ = 0.71073 Å
Tetragonal, *P*4_1_	Cell parameters from 5313 reflections
Hall symbol: P 4w	θ = 2.2–20.7°
*a* = 11.076 (2) Å	µ = 2.16 mm^−^^1^
*c* = 16.478 (3) Å	*T* = 293 K
*V* = 2021.4 (7) Å^3^	Block, yellow
*Z* = 4	0.30 × 0.25 × 0.20 mm
*F*(000) = 944	

### Data collection

**Table:**

Bruker Kappa APEXII CCD diffractometer	4005 independent reflections
Radiation source: fine-focus sealed tube	2553 reflections with *I* > 2σ(*I*)
Graphite monochromator	*R*_int_ = 0.072
ω and φ scan	θ_max_ = 26.1°, θ_min_ = 2.2°
Absorption correction: multi-scan (SADABS; Bruker, 2004)	*h* = −13→13
*T*_min_ = 0.563, *T*_max_ = 0.672	*k* = −13→13
20917 measured reflections	*l* = −20→20

### Refinement

**Table:**

Refinement on *F*^2^	Secondary atom site location: difference Fourier map
Least-squares matrix: full	Hydrogen site location: inferred from neighbouring sites
*R*[*F*^2^ > 2σ(*F*^2^)] = 0.033	H-atom parameters constrained
*wR*(*F*^2^) = 0.083	*w* = 1/[σ^2^(*F*_o_^2^) + (0.0349*P*)^2^] where *P* = (*F*_o_^2^ + 2*F*_c_^2^)/3
*S* = 0.89	(Δ/σ)_max_ < 0.001
4005 reflections	Δρ_max_ = 0.20 e Å^−^^3^
255 parameters	Δρ_min_ = −0.27 e Å^−^^3^
1 restraint	Absolute structure: Flack (1983)
Primary atom site location: structure-invariant direct methods	Absolute structure parameter: 0.016 (8)

### Special details

**Table:**

Geometry. All esds (except the esd in the dihedral angle between two l.s. planes) are estimated using the full covariance matrix. The cell esds are taken into account individually in the estimation of esds in distances, angles and torsion angles; correlations between esds in cell parameters are only used when they are defined by crystal symmetry. An approximate (isotropic) treatment of cell esds is used for estimating esds involving l.s. planes.
Refinement. Refinement of F^2^ against ALL reflections. The weighted R-factor wR and goodness of fit S are based on F^2^, conventional R-factors R are based on F, with F set to zero for negative F^2^. The threshold expression of F^2^ > 2sigma(F^2^) is used only for calculating R-factors(gt) etc. and is not relevant to the choice of reflections for refinement. R-factors based on F^2^ are statistically about twice as large as those based on F, and R- factors based on ALL data will be even larger.

### Fractional atomic coordinates and isotropic or equivalent isotropic displacement parameters (Å^2^)

**Table:**

	*x*	*y*	*z*	*U*_iso_*/*U*_eq_	
Br1	0.00718 (4)	−0.08038 (4)	0.19956 (3)	0.07083 (18)	
S1	0.35274 (8)	0.13083 (9)	0.18370 (7)	0.0532 (3)	
O1	0.3126 (3)	0.1885 (3)	0.10598 (17)	0.0729 (9)	
O2	−0.0067 (3)	0.2442 (3)	0.16015 (19)	0.0668 (9)	
O3	0.1395 (2)	0.3262 (2)	0.39477 (18)	0.0509 (7)	
O4	0.2319 (2)	0.4801 (2)	0.33087 (14)	0.0433 (7)	
N1	0.1482 (2)	0.1911 (2)	0.24983 (18)	0.0340 (7)	
C2	0.3557 (3)	0.2540 (3)	0.2593 (3)	0.0478 (11)	
C3	0.2217 (3)	0.2991 (3)	0.2603 (2)	0.0355 (9)	
H3	0.2088	0.3525	0.2137	0.043*	
C5	0.2117 (3)	0.0786 (3)	0.2304 (2)	0.0393 (10)	
H5	0.2207	0.0213	0.2753	0.047*	
C6	0.1117 (3)	0.0491 (3)	0.1688 (2)	0.0433 (10)	
H6	0.1423	0.0410	0.1132	0.052*	
C7	0.0661 (3)	0.1762 (3)	0.1875 (3)	0.0429 (9)	
C8	0.3934 (4)	0.1997 (4)	0.3415 (3)	0.0725 (15)	
H8A	0.4010	0.2630	0.3809	0.109*	
H8B	0.4695	0.1591	0.3357	0.109*	
H8C	0.3333	0.1431	0.3591	0.109*	
C9	0.4420 (4)	0.3490 (4)	0.2290 (4)	0.090 (2)	
H9A	0.4384	0.4183	0.2640	0.135*	
H9B	0.4200	0.3722	0.1749	0.135*	
H9C	0.5226	0.3172	0.2290	0.135*	
C10	0.1903 (3)	0.3669 (3)	0.3367 (2)	0.0385 (9)	
C11	0.2093 (3)	0.5603 (3)	0.4013 (2)	0.0392 (9)	
H11	0.2150	0.5123	0.4511	0.047*	
C12	0.0850 (3)	0.6124 (3)	0.3960 (3)	0.0385 (9)	
C13	0.0227 (4)	0.6223 (4)	0.3241 (3)	0.0489 (11)	
H13	0.0566	0.5940	0.2761	0.059*	
C14	−0.0922 (4)	0.6754 (4)	0.3235 (3)	0.0641 (13)	
H14	−0.1342	0.6831	0.2749	0.077*	
C15	−0.1417 (4)	0.7151 (4)	0.3933 (4)	0.0660 (13)	
H15	−0.2187	0.7483	0.3925	0.079*	
C16	−0.0808 (4)	0.7075 (4)	0.4651 (3)	0.0628 (13)	
H16	−0.1156	0.7363	0.5127	0.075*	
C17	0.0334 (4)	0.6563 (3)	0.4664 (3)	0.0508 (11)	
H17	0.0756	0.6515	0.5150	0.061*	
C18	0.3106 (3)	0.6508 (3)	0.4006 (2)	0.0370 (9)	
C19	0.3129 (4)	0.7455 (4)	0.3469 (3)	0.0479 (11)	
H19	0.2509	0.7545	0.3094	0.057*	
C20	0.4071 (4)	0.8277 (4)	0.3484 (3)	0.0542 (12)	
H20	0.4083	0.8914	0.3116	0.065*	
C21	0.4986 (4)	0.8154 (4)	0.4038 (3)	0.0568 (12)	
H21	0.5617	0.8708	0.4047	0.068*	
C22	0.4969 (3)	0.7212 (4)	0.4581 (3)	0.0597 (12)	
H22	0.5587	0.7129	0.4959	0.072*	
C23	0.4040 (3)	0.6394 (3)	0.4562 (3)	0.0486 (10)	
H23	0.4036	0.5755	0.4928	0.058*	

### Atomic displacement parameters (Å^2^)

**Table:**

	*U*^11^	*U*^22^	*U*^33^	*U*^12^	*U*^13^	*U*^23^
Br1	0.0570 (3)	0.0518 (3)	0.1037 (4)	−0.0182 (2)	0.0112 (3)	−0.0148 (3)
S1	0.0352 (5)	0.0467 (6)	0.0777 (9)	−0.0011 (4)	0.0143 (6)	−0.0181 (6)
O1	0.075 (2)	0.090 (2)	0.054 (2)	−0.0136 (18)	0.0229 (18)	−0.0001 (18)
O2	0.0562 (19)	0.0591 (19)	0.085 (2)	0.0144 (16)	−0.0230 (16)	0.0023 (17)
O3	0.0619 (18)	0.0393 (16)	0.0516 (17)	−0.0119 (13)	0.0178 (16)	−0.0062 (14)
O4	0.0485 (15)	0.0305 (14)	0.0509 (17)	−0.0064 (12)	0.0093 (13)	−0.0076 (12)
N1	0.0290 (16)	0.0246 (16)	0.0486 (19)	0.0025 (12)	0.0027 (15)	−0.0078 (14)
C2	0.030 (2)	0.043 (2)	0.070 (3)	−0.0048 (19)	0.012 (2)	−0.015 (2)
C3	0.035 (2)	0.0265 (19)	0.046 (2)	−0.0021 (16)	0.0077 (18)	−0.0008 (17)
C5	0.039 (2)	0.032 (2)	0.047 (3)	0.0025 (17)	0.0037 (18)	−0.0097 (17)
C6	0.043 (2)	0.038 (2)	0.049 (3)	−0.0055 (17)	0.0026 (19)	−0.0081 (19)
C7	0.042 (2)	0.034 (2)	0.053 (3)	−0.0004 (17)	−0.001 (2)	−0.002 (2)
C8	0.052 (3)	0.083 (4)	0.082 (4)	0.016 (2)	−0.023 (3)	−0.030 (3)
C9	0.058 (3)	0.061 (3)	0.151 (6)	−0.027 (3)	0.045 (3)	−0.033 (3)
C10	0.033 (2)	0.030 (2)	0.053 (3)	−0.0017 (17)	0.002 (2)	−0.009 (2)
C11	0.048 (2)	0.032 (2)	0.038 (2)	−0.0027 (17)	0.0054 (19)	−0.0052 (18)
C12	0.039 (2)	0.030 (2)	0.046 (2)	−0.0059 (16)	0.000 (2)	−0.0033 (18)
C13	0.044 (2)	0.047 (2)	0.056 (3)	−0.0034 (19)	0.000 (2)	−0.011 (2)
C14	0.046 (3)	0.063 (3)	0.083 (4)	−0.006 (2)	−0.015 (3)	−0.010 (3)
C15	0.047 (3)	0.044 (3)	0.107 (4)	0.001 (2)	−0.001 (3)	−0.007 (3)
C16	0.061 (3)	0.049 (3)	0.079 (4)	0.010 (2)	0.020 (3)	−0.010 (3)
C17	0.061 (3)	0.042 (2)	0.049 (3)	0.007 (2)	0.005 (2)	−0.007 (2)
C18	0.033 (2)	0.032 (2)	0.046 (2)	0.0010 (16)	−0.0013 (18)	−0.0067 (19)
C19	0.040 (2)	0.048 (3)	0.057 (3)	−0.0060 (19)	−0.006 (2)	0.002 (2)
C20	0.051 (3)	0.045 (2)	0.066 (3)	−0.008 (2)	0.008 (2)	−0.002 (2)
C21	0.039 (2)	0.056 (3)	0.075 (3)	−0.003 (2)	0.003 (2)	−0.021 (3)
C22	0.042 (2)	0.053 (3)	0.085 (3)	0.004 (2)	−0.019 (2)	−0.009 (3)
C23	0.043 (2)	0.037 (2)	0.065 (3)	0.0060 (17)	−0.011 (2)	0.001 (2)

### Geometric parameters (Å, º)

**Table:**

Br1—C6	1.912 (4)	C11—C12	1.495 (5)
S1—O1	1.499 (3)	C11—C18	1.505 (5)
S1—C5	1.835 (4)	C11—H11	0.9800
S1—C2	1.847 (4)	C12—C13	1.375 (6)
O2—C7	1.191 (4)	C12—C17	1.382 (5)
O3—C10	1.199 (4)	C13—C14	1.401 (6)
O4—C10	1.338 (4)	C13—H13	0.9300
O4—C11	1.484 (4)	C14—C15	1.348 (7)
N1—C7	1.382 (5)	C14—H14	0.9300
N1—C3	1.458 (4)	C15—C16	1.364 (6)
N1—C5	1.466 (4)	C15—H15	0.9300
C2—C9	1.506 (6)	C16—C17	1.386 (6)
C2—C8	1.540 (6)	C16—H16	0.9300
C2—C3	1.567 (5)	C17—H17	0.9300
C3—C10	1.505 (5)	C18—C19	1.372 (5)
C3—H3	0.9800	C18—C23	1.388 (5)
C5—C6	1.537 (5)	C19—C20	1.385 (6)
C5—H5	0.9800	C19—H19	0.9300
C6—C7	1.528 (5)	C20—C21	1.371 (6)
C6—H6	0.9800	C20—H20	0.9300
C8—H8A	0.9600	C21—C22	1.374 (6)
C8—H8B	0.9600	C21—H21	0.9300
C8—H8C	0.9600	C22—C23	1.371 (5)
C9—H9A	0.9600	C22—H22	0.9300
C9—H9B	0.9600	C23—H23	0.9300
C9—H9C	0.9600		
			
O1—S1—C5	103.90 (17)	O3—C10—O4	124.8 (3)
O1—S1—C2	105.5 (2)	O3—C10—C3	126.0 (3)
C5—S1—C2	88.00 (16)	O4—C10—C3	109.1 (3)
C10—O4—C11	116.5 (3)	O4—C11—C12	109.9 (3)
C7—N1—C3	123.6 (3)	O4—C11—C18	105.5 (3)
C7—N1—C5	93.0 (3)	C12—C11—C18	115.4 (3)
C3—N1—C5	117.1 (3)	O4—C11—H11	108.6
C9—C2—C8	113.1 (4)	C12—C11—H11	108.6
C9—C2—C3	112.4 (3)	C18—C11—H11	108.6
C8—C2—C3	111.8 (3)	C13—C12—C17	119.2 (4)
C9—C2—S1	107.7 (3)	C13—C12—C11	122.9 (4)
C8—C2—S1	108.0 (3)	C17—C12—C11	117.9 (4)
C3—C2—S1	103.1 (3)	C12—C13—C14	119.7 (4)
N1—C3—C10	112.3 (3)	C12—C13—H13	120.1
N1—C3—C2	105.4 (3)	C14—C13—H13	120.1
C10—C3—C2	112.8 (3)	C15—C14—C13	120.0 (4)
N1—C3—H3	108.7	C15—C14—H14	120.0
C10—C3—H3	108.7	C13—C14—H14	120.0
C2—C3—H3	108.7	C14—C15—C16	121.3 (4)
N1—C5—C6	88.8 (3)	C14—C15—H15	119.4
N1—C5—S1	103.4 (2)	C16—C15—H15	119.4
C6—C5—S1	113.8 (3)	C15—C16—C17	119.3 (4)
N1—C5—H5	115.7	C15—C16—H16	120.4
C6—C5—H5	115.7	C17—C16—H16	120.4
S1—C5—H5	115.7	C12—C17—C16	120.6 (4)
C7—C6—C5	84.8 (3)	C12—C17—H17	119.7
C7—C6—Br1	115.9 (3)	C16—C17—H17	119.7
C5—C6—Br1	114.8 (3)	C19—C18—C23	118.8 (4)
C7—C6—H6	112.8	C19—C18—C11	121.8 (3)
C5—C6—H6	112.8	C23—C18—C11	119.4 (3)
Br1—C6—H6	112.8	C18—C19—C20	120.4 (4)
O2—C7—N1	130.6 (3)	C18—C19—H19	119.8
O2—C7—C6	136.9 (4)	C20—C19—H19	119.8
N1—C7—C6	92.4 (3)	C21—C20—C19	120.2 (4)
C2—C8—H8A	109.5	C21—C20—H20	119.9
C2—C8—H8B	109.5	C19—C20—H20	119.9
H8A—C8—H8B	109.5	C20—C21—C22	119.9 (4)
C2—C8—H8C	109.5	C20—C21—H21	120.0
H8A—C8—H8C	109.5	C22—C21—H21	120.0
H8B—C8—H8C	109.5	C23—C22—C21	119.8 (4)
C2—C9—H9A	109.5	C23—C22—H22	120.1
C2—C9—H9B	109.5	C21—C22—H22	120.1
H9A—C9—H9B	109.5	C22—C23—C18	120.9 (4)
C2—C9—H9C	109.5	C22—C23—H23	119.5
H9A—C9—H9C	109.5	C18—C23—H23	119.5
H9B—C9—H9C	109.5		
			
O1—S1—C2—C9	−58.9 (3)	C5—C6—C7—N1	−7.5 (3)
C5—S1—C2—C9	−162.8 (3)	Br1—C6—C7—N1	107.6 (3)
O1—S1—C2—C8	178.6 (3)	C11—O4—C10—O3	−1.9 (5)
C5—S1—C2—C8	74.7 (3)	C11—O4—C10—C3	−179.8 (3)
O1—S1—C2—C3	60.1 (3)	N1—C3—C10—O3	20.8 (5)
C5—S1—C2—C3	−43.8 (3)	C2—C3—C10—O3	−98.2 (4)
C7—N1—C3—C10	114.4 (4)	N1—C3—C10—O4	−161.3 (3)
C5—N1—C3—C10	−131.4 (3)	C2—C3—C10—O4	79.7 (4)
C7—N1—C3—C2	−122.4 (4)	C10—O4—C11—C12	−82.9 (4)
C5—N1—C3—C2	−8.2 (4)	C10—O4—C11—C18	152.0 (3)
C9—C2—C3—N1	152.1 (4)	O4—C11—C12—C13	−23.0 (5)
C8—C2—C3—N1	−79.5 (4)	C18—C11—C12—C13	96.2 (4)
S1—C2—C3—N1	36.3 (3)	O4—C11—C12—C17	159.5 (3)
C9—C2—C3—C10	−85.0 (4)	C18—C11—C12—C17	−81.4 (4)
C8—C2—C3—C10	43.4 (4)	C17—C12—C13—C14	−0.7 (6)
S1—C2—C3—C10	159.2 (3)	C11—C12—C13—C14	−178.2 (4)
C7—N1—C5—C6	−7.8 (3)	C12—C13—C14—C15	−0.8 (6)
C3—N1—C5—C6	−138.3 (3)	C13—C14—C15—C16	1.6 (7)
C7—N1—C5—S1	106.4 (2)	C14—C15—C16—C17	−0.9 (7)
C3—N1—C5—S1	−24.1 (4)	C13—C12—C17—C16	1.3 (6)
O1—S1—C5—N1	−66.7 (3)	C11—C12—C17—C16	179.0 (4)
C2—S1—C5—N1	38.7 (3)	C15—C16—C17—C12	−0.6 (6)
O1—S1—C5—C6	28.0 (3)	O4—C11—C18—C19	76.6 (4)
C2—S1—C5—C6	133.5 (3)	C12—C11—C18—C19	−45.0 (5)
N1—C5—C6—C7	7.1 (3)	O4—C11—C18—C23	−104.1 (4)
S1—C5—C6—C7	−97.1 (3)	C12—C11—C18—C23	134.3 (4)
N1—C5—C6—Br1	−109.1 (3)	C23—C18—C19—C20	0.2 (6)
S1—C5—C6—Br1	146.69 (19)	C11—C18—C19—C20	179.5 (4)
C3—N1—C7—O2	−43.8 (6)	C18—C19—C20—C21	−0.4 (6)
C5—N1—C7—O2	−169.4 (5)	C19—C20—C21—C22	0.1 (6)
C3—N1—C7—C6	133.5 (3)	C20—C21—C22—C23	0.3 (6)
C5—N1—C7—C6	7.9 (3)	C21—C22—C23—C18	−0.5 (6)
C5—C6—C7—O2	169.5 (5)	C19—C18—C23—C22	0.2 (6)
Br1—C6—C7—O2	−75.4 (6)	C11—C18—C23—C22	−179.1 (4)

### Hydrogen-bond geometry (Å, º)

*Cg* is the centroid of the C18–C23 ring.

**Table:**

*D*—H···*A*	*D*—H	H···*A*	*D*···*A*	*D*—H···*A*
C21—H21···O2^i^	0.93	2.54	3.465 (5)	171
C22—H22···O1^ii^	0.93	2.55	3.375 (5)	148
C14—H14···*Cg*^iii^	0.93	2.68	3.533 (5)	153

Symmetry codes: (i) −*y*+1, *x*+1, *z*+1/4; (ii) −*x*+1, −*y*+1, *z*+1/2; (iii) *y*−1, −*x*+1, *z*−1/4.
